# Expression of radial glial markers (GFAP, BLBP and GS) during telencephalic development in the catshark (*Scyliorhinus canicula*)

**DOI:** 10.1007/s00429-018-1758-2

**Published:** 2018-09-21

**Authors:** A. Docampo-Seara, G. N. Santos-Durán, E. Candal, Miguel Ángel Rodríguez Díaz

**Affiliations:** 1 Departamento de Bioloxía Funcional, Centro de Investigación en Bioloxía (CIBUS), Universidade de Santiago de Compostela, 15782 Santiago de Compostela, Spain; 2 Laboratory of Artificial and Natural Evolution (LANE), Department of Genetics and Evolution, University of Geneva, Geneva, Switzerland

**Keywords:** Radial glia, Oligodendrocytes, Proliferation, Development, GFAP, BLBP, GS

## Abstract

Radial glial cells (RGCs) are the first cell populations of glial nature to appear during brain ontogeny. They act as primary progenitor (stem) cells as well as a scaffold for neuronal migration. The proliferative capacity of these cells, both in development and in adulthood, has been subject of interest during past decades. In contrast with mammals where RGCs are restricted to specific ventricular areas in the adult brain, RGCs are the predominant glial element in fishes. However, developmental studies on the RGCs of cartilaginous fishes are scant. We have studied the expression patterns of RGCs markers including glial fibrillary acidic protein (GFAP), brain lipid binding protein (BLBP), and glutamine synthase (GS) in the telencephalic hemispheres of catshark (*Scyliorhinus canicula*) from early embryos to post-hatch juveniles. GFAP, BLBP and GS are first detected, respectively, in early, intermediate and late embryos. Expression of these glial markers was observed in cells with radial glia morphology lining the telencephalic ventricles, as well as in their radial processes and endfeet at the pial surface and their expression continue in ependymal cells (or tanycytes) in early juveniles. In addition, BLBP- and GS-immunoreactive cells morphologically resembling oligodendrocytes were observed. In late embryos, most of the GFAP- and BLBP-positive RGCs also coexpress GS and show proliferative activity. Our results indicate the existence of different proliferating subpopulations of RGCs in the embryonic ventricular zone of catshark. Further investigations are needed to determine whether these proliferative RGCs could act as neurogenic and/or gliogenic precursors.

**Electronic supplementary material:**

The online version of this article (10.1007/s00429-018-1758-2) contains supplementary material, which is available to authorized users.

## Introduction

The telencephalon is one of the most studied areas in the entire central nervous system (CNS) since it develops into various structures that are responsible of complex functions such as cognition, memory or development of social skills. Neurodegenerative diseases affecting these areas have become a concern, since the mechanisms for neuronal loss are intricate and effective treatments for these diseases do not exist. In the last decades, the identification of different types of neural stem cells in the brain together with the comprehension of how they produce the impressive diversity of brain cells has become crucial for developing cellular therapies for brain repair (Kriegstein and Alvarez-Buylla 2009).

During development, neuroepithelial cells (NECs) are the primary neural stem cells in the CNS (see Falk and Götz 2017 and references therein). In the embryonic mouse forebrain, with the onset of neurogenesis (E9–E10), NECs present in the neural tube give rise to radial glial cells (RGCs), the first cell type of glial nature arising in the developing central nervous system (for review see: Götz and Huttner 2005; Alvarez-Buylla and Kriegstein 2013; Götz 2013; Turrero and Harwell 2017). These cells were first described in the nineteenth century in the embryonic spinal cord and cerebral cortex by Camillo Golgi and Giussepe Magini, respectively (for review see: Bentivoglio and Mazzarello 1999; Kriegstein and Götz 2003; Farkas and Huttner 2008; Götz 2013). They are characterized by a bipolar morphology with a ventricular contacting body and a radial process ending in a subpial endfoot. Ultrastructural studies during corticogenesis carried out by Rakic (1972) showed that radial processes of RGCs act as a scaffold for migration of new-born neurons to upper cortical layers. Later studies have shown that RGCs are involved in the correct patterning of the central nervous system (for review see: Götz 2013). Importantly, numerous studies on the developing mammalian telencephalon have additionally shown that RGCs also act as neural stem cells since they are the origin of all neurons and the different subtypes of differentiated glial cells (astrocytes, oligodendrocytes and ependymal cells), either directly or through other neural progenitor cell types subsequently derived from them (Götz et al. 1998; Marshall et al. 2003; Spassky et al. 2005; Alvarez-Buylla and Kriegstein 2013; Götz 2013; Taverna et al. 2014; Beattie and Hippenmeyer 2017; Turrero and Harwell 2017). RGCs also act as progenitor cells in the developing telencephalon of non-mammalian vertebrate groups including reptiles (Clinton et al. 2014; Martínez-Cerdeño et al. 2016) amphibians (D’Amico et al. 2011; Moreno and González 2017) and teleosts (Lyons et al. 2003; Alexandre et al. 2010).

In mammals, the vast majority of RGCs disappear in postnatal stages, except for a few locations such as the lateral telencephalic ventricles, hypothalamus and cerebellum where radial glia persists throughout life (Malatesta et al. 2008; Götz 2013). In non-mammalian vertebrates RGCs that act as progenitors are also persistent in some areas of the brain. Proliferating RGC have been detected in the telencephalon of birds (Alvarez-Buylla et al. 1990, 1998), reptiles (reviewed by González-Granero et al. 2011; Clinton et al. 2014), amphibians (Kirkham et al. 2014) and teleost fishes (März et al. 2010). RGCs (named ependymoglia) have been studied in the brain of lampreys (Merrick et al. 1995). However, no proliferation has been detected in the adult telencephalon (Villar-Cheda et al. 2006).

Therefore, the term “glial cell” can result puzzling since it includes both these progenitor RGCs, as well as a differentiated population of parenchymal astrocytes, oligodendrocytes, and ependymal cells, with which it shares some properties (Kriegstein and Alvarez-Buylla 2009). In this context, various markers have been commonly used to identify RGCs, including the brain lipid binding protein (BLBP), the intermediate filament glial fibrillary acidic protein (GFAP), and the enzyme glutamine synthase (GS). BLBP is a protein involved in lipid metabolism and membrane synthesis. Its expression has been related with the neurogenic and/or gliogenic potential of radial glia (Hartfuss et al. 2001; Pinto and Götz 2007; Podgornyi and Aleksandrova 2009; März et al. 2010; Li et al. 2011; Diotel et al. 2016). Some studies have referred to BLBP expression in the embryonic and adult brain in non-mammalian vertebrates. This protein has been detected in RGCs in the telencephalon of amphibian embryos (D’Amico et al. 2011; Moreno and González 2017) and adult zebrafish (Grupp et al. 2010; März et al. 2010; Diotel et al. 2016). However, BLBP does not allow to clearly distinguish RGCs from NECs since it is expressed by both cell types (Pinto and Götz 2007; Götz 2013; Than-Trong and Bally-Cuif 2015; Diotel et al. 2016). On the other hand, in mammals, glial markers like GFAP and GS are expressed in RGCs but not in NECs, allowing distinguishing between these types of neural stem cells. However, these markers are also expressed by differentiated glia including astroglia and ependymal cells (for review see: Götz 2013).

In summary, in mammals and other non-mammalian vertebrates, different types of progenitor cells express similar markers that are additionally shared with several types of differentiated glial cells (Than-Trong and Bally-Cuif 2015). Therefore, a good comprehension of the emergence/development of cells with hallmarks of radial glial and a deep characterization of these cells is necessary. Cartilaginous fishes occupy a key phylogenetic position in the tree of jawed vertebrates as the sister group of bony vertebrates, which include bony fishes and tetrapods (Coolen et al. 2009). Our aim is to investigate and characterize progenitor RGCs in the telencephalon of the lesser spotted dogfish (catshark) *Scyliorhinus canicula*, a model species of cartilaginous fishes for evo-devo studies (Coolen et al. 2009; Rodríguez-Moldes et al. 2017). With this aim, we analysed the expression of three RGC molecular markers, GFAP, BLBP and GS in the telencephalic hemispheres in embryos and early juveniles using immunohistochemical and double immunofluorescence techniques. We additionally used double immunofluorescence techniques combining these glial markers with the proliferating cell nuclear antigen (PCNA) to evaluate the proliferative potential of different subtypes of RGCs.

## Materials and methods

### Experimental animals

We used 15 embryos of *S. canicula* from stages 25 (S25) to 33 (S33) and 3 posthatching juveniles. Most embryos were provided by the Marine Biological Model Supply Service of the CNRS, UPMC Roscoff Biological Station (France) and some embryos and juveniles were kindly provided by the aquarium of O Grove (Galicia, Spain). Embryos were staged by their external features according to Ballard et al. (1993). Eggs were raised in seawater tanks under standard conditions of temperature (15–16 °C), pH (7.5–8.5) and salinity (35 g/L) and suitable measures were taken to minimize animal pain and discomfort. All procedures conformed to the guidelines established by the European Communities Council Directive of 22 September 2010 (2010/63/UE) and by Spanish Royal Decree 53/2013 for animal experimentation, and were approved by the Ethics Committee of the University of Santiago de Compostela.

### Tissue processing

Embryos were deeply anesthetized with 0.5% tricaine methane sulfonate (MS- 222; Sigma, St. Louis, MO, USA) in seawater and separated from the yolk before fixation in 4% paraformaldehyde (PFA) in elasmobranch’s phosphate buffer [EPB: 0.1 M phosphate buffer (PB) containing 1.75% of urea, pH 7.4] for 48–72 h depending on the stage of development. Sharks from stage 32 (S32) onwards were deeply anesthetized with MS-222 and then perfused intracardially with elasmobranch Ringer’s solution (see Ferreiro-Galve et al. 2012) followed by 4% PFA in EPB. Brains were removed and postfixed in the same fixative for 24–48 h at 4 °C. Subsequently, they were rinsed in PB saline (PBS), cryoprotected with 30% sucrose in PB, embedded in OCT compound (Tissue Tek, Torrance, CA), and frozen with liquid nitrogen-cooled isopentane. Parallel series of sections (16–18 µm thick) were obtained in transverse or sagittal planes on a cryostat and mounted on to Superfrost Plus (Menzel-Glasser, Madison, WI, USA) slides.

### Immunohistochemistry

Sections were pre-treated with 0.01 M citrate buffer pH 6.0 for 30 min at 90 °C for heat-induced epitope retrieval and allowed to cool for 15 min at room temperature (RT). Sections were rinsed in 0.05 M Tris-buffered saline (TBS; pH 7.4) for 5 min and treated with 10% H_2_O_2_ in TBS for 30 min at RT to block endogenous peroxidase activity. Sections were rinsed in 0.05 M TBS pH 7.4 for 5 min, and incubated approximately for 15 h at RT with primary antibodies (see Table 1). Sections were rinsed three times in 0.05 M TBS pH 7.4 for 10 min each, and incubated in the appropriate HRP-coupled secondary antibody (see Table 1) for 1 h at RT. All dilutions were made with TBS containing 15% normal goat serum (Millipore, Billerica, MA, USA) 0.2% Triton X-100 (Sigma) and 2% bovine serum albumin (BSA, Sigma). All incubations were carried out in a humid chamber. Then, sections were rinsed three times in 0.05 M TBS pH 7.4 for 10 min each. The immunoreaction was developed with 0.25 mg/ml diaminobenzidine tetrahydrochloride (DAB, Sigma) in TBS pH 7.4 and 0.00075% H_2_O_2_, or with SIGMAFAST™ 3.3-DAB tablets as indicated by the manufacturer. To enhance the GFAP immunoreaction in sections of early developmental stages, 2.5 mg/ml nickel ammonium sulphate was added. Finally, the sections were dehydrated and coverslipped. Additional information about the primary and secondary antibodies is included in Table 1.

**Table 1:** Primary and secondary antibodies used

Primary antibody	Source	Working dilution	Secondary antibody	Source	Working dilution
GFAP	Polyclonal rabbit anti-GFAP Dako Cat. Nº. Z033429	1:500	Goat anti-rabbit HRP coupled	Dako, Glostrup, Denmark	1:200
GS	Monoclonal mouse anti-GS Millipore Cat. Nº. MAB302	1:500	Goat anti-mouse HRP coupled	Dako, Glostrup, Denmark	1:200
BLBP	Polyclonal rabbit anti-BLBP Millipore Cat. Nº. ABN14	1:300	488-conjugated donkey anti-mouse	Alexa Fluor Molecular Probes, Eugene, OR	1:200
PCNA	Monoclonal mouse anti-PCNA Sigma Cat. Nº. P8825	1:500	546-conjugated donkey anti-rabbit	Alexa Fluor Molecular Probes, Eugene, OR	1:200
PH3	Polyclonal rabbit anti-PH3 Millipore 06-570	1:300			

### Control and specificity of antibodies

**Fig. 1:**
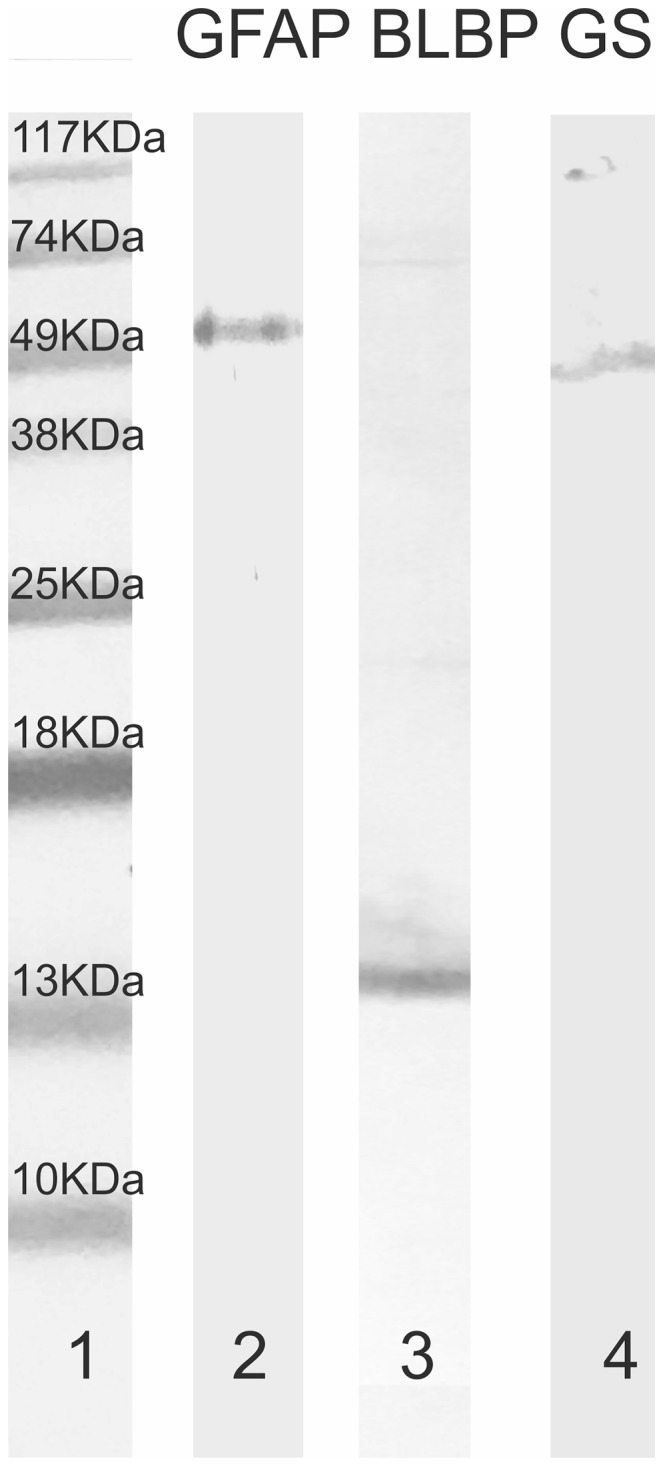
Immunoblots of SDS–polyacrylamide gel of *S. canicula* adult brain protein extracts stained by anti-GFAP (lane 2), anti-BLBP (lane 3), and anti-GS (lane 4) antibodies. GFAP lane showed single a band of around 49 kDa. BLBP lane showed a single band of around 13 kDa. GS lane showed a single band between 38 and 49 kDa

The monoclonal antibody against the proliferating cell nuclear antigen (PCNA) has been previously used in our lab to label progenitor cells in the brain, retina and olfactory system of the catshark or lesser spotted dogfish (Quintana-Urzainqui et al. 2014, 2015; Sánchez-Farías and Candal 2015, among others). PCNA is present in proliferating cells along the entire cell cycle, though its expression is stronger during the S phase (Zerjatke et al. 2017). The anti-PH3 antibody has been used previously as a marker of mitotic cells in the telencephalon of *S. canicula* (Quintana-Urzainqui et al. 2015). The polyclonal antibody against GFAP has been previously used as marker of glial cells in the brain and retina of *S. canicula* (Quintana-Urzainqui et al. 2014, 2015; Sánchez-Farías and Candal 2016). The monoclonal antibody against GS has been previously used as a marker of mature Müller cells in the retina of *S. canicula* (Bejarano-Escobar et al. 2012; Sánchez-Farías and Candal 2016). On the other hand, the BLBP antibody was never characterized in sharks. The specificity of all antibodies against glial markers used in this work was tested by Western Blot analysis of brain protein extracts of adult catshark using standard procedures (for further information of methods see Anadón et al. 2000). ProSieve proteins standards (Lonza, Rockland, ME) were used as molecular weight markers (Fig. 1, lane 1). As positive controls, protein extracts of adult mouse brain were run in parallel to assess the specificity of antibodies.

According to the manufacturer, the GFAP antibody recognizes a 50 kDa intracytoplasmic filamentous protein characteristic of astrocytes. In blots of shark protein extracts, it recognizes a single band around 49 kDa (Fig. 1, lane 2). With regard to BLBP, according to the manufacturer the antibody labels a band about 15 kDa in western blot analysis in mouse brain protein extracts. In the present blot, a band about 15 kDa can be recognizable (Fig. 1, lane 3). In the case of GS, in mouse brain extracts of proteins, a band of approximately 44 kDa can be detected by western blot. In this blot a band close to 49 kDa can be observed.

### Image acquisition and analysis

Light field images were obtained with an Olympus BX51 microscope equipped with an Olympus DP71 colour digital camera (Olympus, Tokyo, Japan). Fluorescent sections were analysed and photographed with a spectral laser scanning microscope (TCS-SP2, Leica, Wetzlar, Germany) using a combination of blue and green excitation lasers. Stacks of confocal images were acquired separately for each laser channel with steps of 0.8 µm or 2 µm along the *z*-axis. *Z*-projections of an average of 12 optical sections were done with LITE software (Leica). Photographs were adjusted for brightness and contrast, and plates were prepared using Corel Draw X3.

### Cell counting

Cell counting was performed attending to immunoreactivity both in cell bodies and processes. To estimate the amount of single and double-labelled cells for glial markers (GFAP/GS and BLBP/GS), cells were counted in two regions of the pallium (dorsomedial and presumptive ventral pallium) and in the subpallium. For quantification, confocal images from central stacks of 3–4 µm were taken and cells from areas of 50 × 50 µm of the ventricle were counted manually. One area in each region was counted in two different sections from two different S32/33 embryos (4 area/region/experiment). Average, standard deviations and bar representations and the proportion of each cell type were determined using Microsoft Excel 2016. For further information about numbers and graphics see Supp. Fig. 1.

## Results

In this study, we investigated the expression pattern of GFAP, BLBP and GS in a series of catshark embryos representative of the early (S25–S26), middle (S27–S31) and late (S32–S34) periods of telencephalic development (Ballard et al. 1993). In the telencephalon, the middle period corresponds with early (S28) to middle (S31) stages of neurogenesis. In S29 embryos, the telencephalic walls mainly consist of a pseudostratified neuroepithelium. Pallial and subpallial regions are already recognizable but no further subdivisions or differences along the rostro-caudal axis of the telencephalon are appreciable. By S31, the zonation of the telencephalic walls into ventricular (neuroepithelial), intermediate and marginal zones (from the ventricle to the meningeal surface) is already recognizable and the major pallial regions are observed. From S32 onwards, important morphological changes take place and the basic mature structure of the telencephalon is progressively achieved. The cytoarchitectonic organization of the telencephalon during this late period is highly similar to that observed in juvenile and adults. The mature telencephalon of *S. canicula* consists of the telencephalic hemispheres and the olfactory bulb. According to the classical view (Smeets et al. 1983) the telencephalic hemispheres can be subdivided in an evaginated portion (rostral and medial telencephalon) and in a non evaginated portion (caudal telencephalon). For further information of the development of telencephalon of dogfish see Quintana-Urzainqui et al. (2015) and Rodríguez-Moldes et al. (2017).

**Fig. 2:**
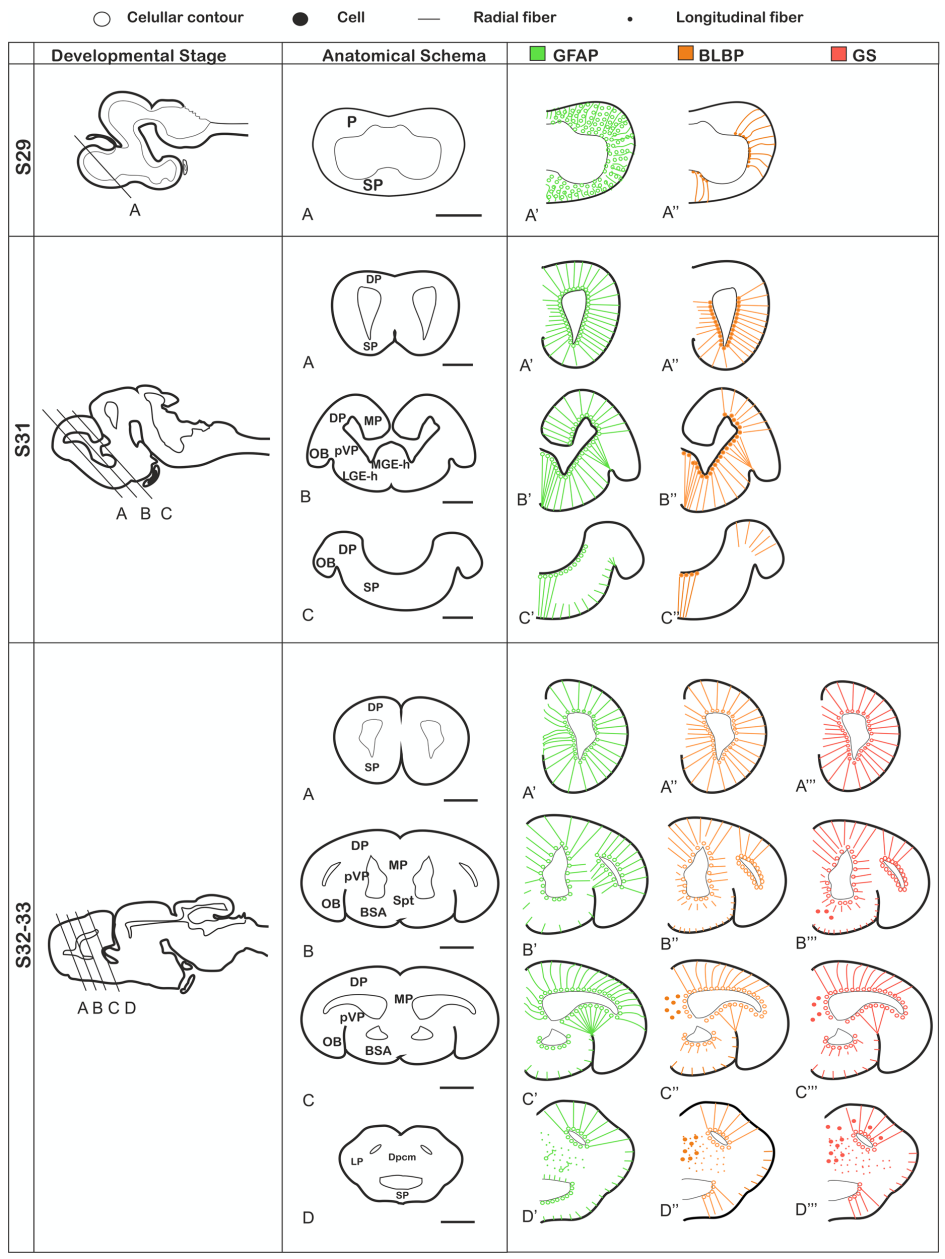
Schema summarizing the expression pattern of GFAP (green color), BLBP (orange color), GS (red color) from S29 to S32. The choroid plexus has not been represented in this schema. Scale bars 200 µm

**Fig. 3:**
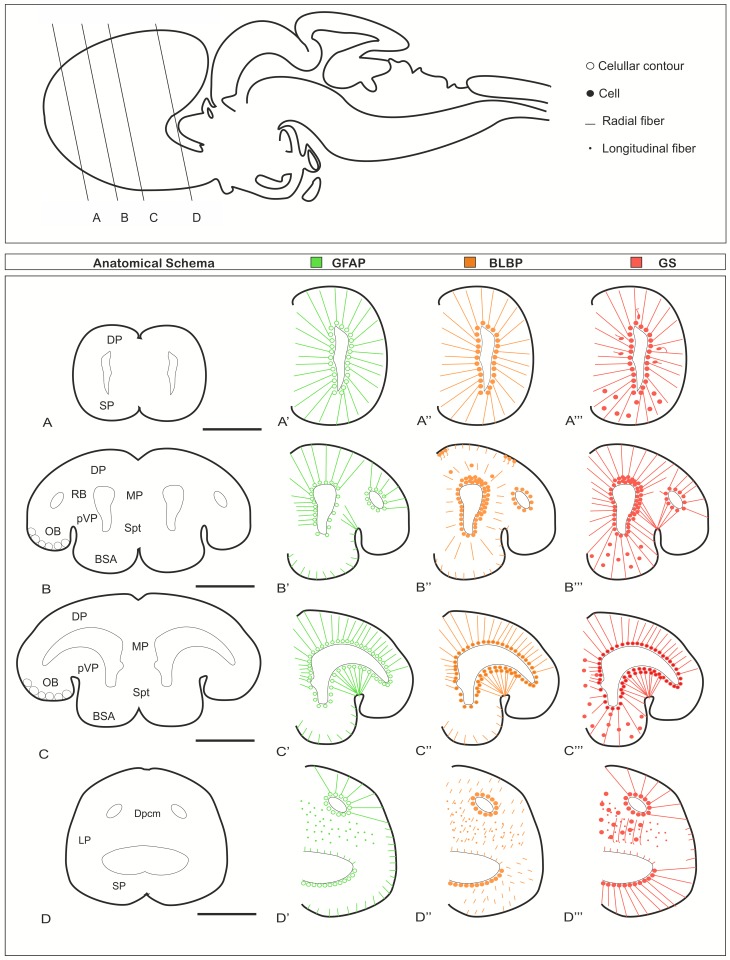
Schema summarizing the expression pattern of GFAP (green color), BLBP (orange color), GS (red color) in posthatching juveniles. The choroid plexus has not been represented in this schema. Scale bars 500 µm

The aims of the present study are to investigate the development of the radial glial system in basal vertebrates and examine the proliferative potential of these cells. The expression pattern of the three glial markers (GFAP, BLBP and GS) and the coexpression study is described below. Schematic drawings about the distribution of GFAP, BLBP and GS expressing cells in the catshark telencephalon during embryonic development and in juveniles are presented in Figs. 2 and 3, respectively.

### Expression pattern of GFAP in the telencephalic hemispheres of embryonic stages and juveniles

**Fig. 4:**
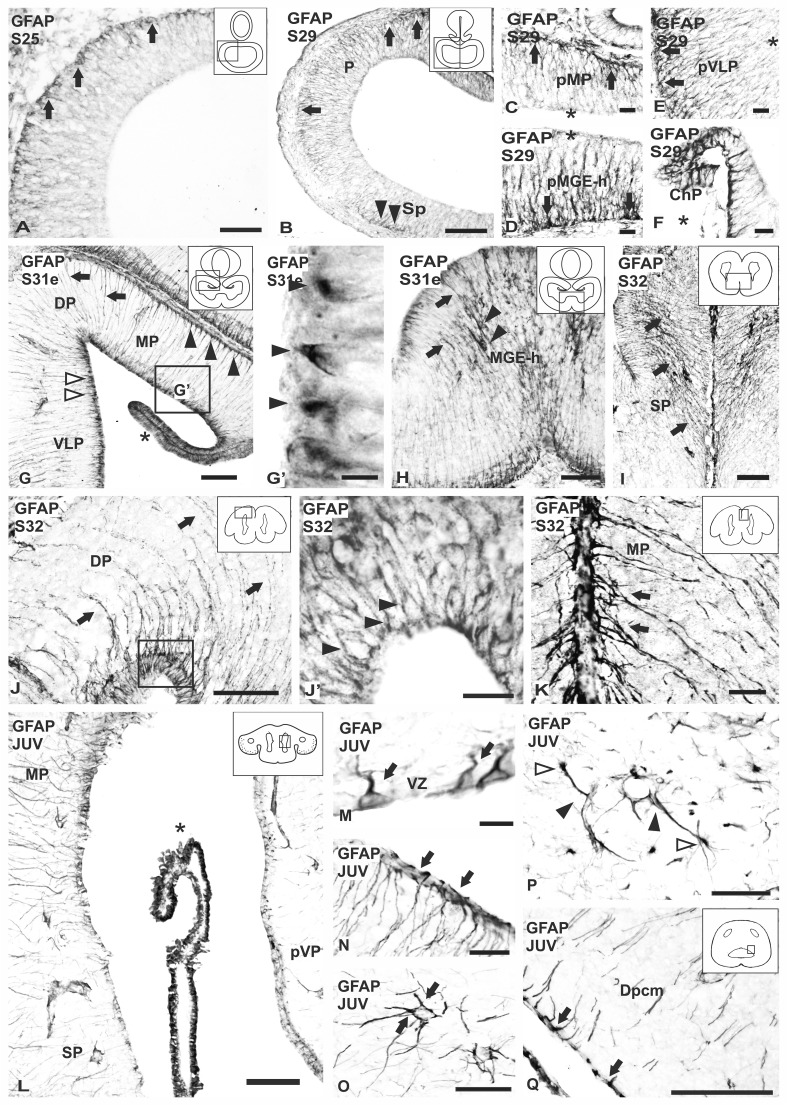
Photomicrographs of transverse sections of the telencephalon of *S. canicula* showing the expression pattern of GFAP in embryos (**a**–**k**) and juveniles (**l**–**q**). **a** Section of the telencephalon of a S25 embryo showing GFAP immunoreactivity in numerous endfeet in the pial surface of pallium (arrows). **b**–**f** Photomicrographs of a S29 embryo. **b** Photomicrograph at lower magnification showing GFAP immunoreactivity in radial processes extending from the ventricular surface to the pia in the pallium (arrows) and subpallium (arrowheads). **c**–**e** Photomicrographs at higher magnification showing an intense GFAP immunolabelling in the midline of the telencephalon (arrows) (**c, d**), in contrast to the rest of the telencephalon which shows weak immunoreactivity (arrows) (**e**). **f** Photomicrograph at higher magnification of the choroid plexus which show an intense GFAP immunoreactivity. **g, h** Photomicrographs from a S31 embryo at different magnifications. **g** Transverse section of the telencephalon showing radial processes (arrows), endfeet (arrowheads) and numerous positive cells lining the ventricular zone (empty arrowheads) positive for GFAP. Note the intense immunoreactivity in the choroid plexus (asterisk). **g**′ Photomicrograph at higher magnification of the ventricular surface showing the basal portion of cells close to the ventricle (arrowheads) immunoreactive to GFAP. **h** Transverse section of the subpallial midline showing intense immunoreactivity for GFAP at the ventricular and pial zone and numerous radial processes positive for GFAP (arrows); cells GFAP positive are intermingled between radial processes (arrowheads). **i**–**k** Photomicrographs from S32 embryos. **i** Section of the rostral telencephalon showing intense convergent processes (arrows) in the midline of subpallium. **j**–**j**′ Photomicrographs at different magnifications of the dorsal pallium showing curvy radial processes (arrows) and numerous cells in the ventricular zone showing GFAP immunoreactivity in the periphery of the cell body (arrowheads in **j**′). **k** Photomicrograph at high magnification of the medial pallium showing intense GFAP positive radial processes converging in the pallial midline (arrows). **l**–**q** Transverse sections of GFAP immunoreactivity in the telencephalon of juveniles. **l** Panoramic view of the ventricular zone of the telencephalon showing differences in the expression of GFAP between medial pallium, presumptive ventral pallium and subpallium. Note strong immunoreactivity in the choroid plexus (asterisk). **m**–**o** Magnifications of the ventricular zone, pial surface and blood vessels, respectively, showing ventricular positive cells (arrows in **m**), endfeet in the pial surface (arrows in **n**) and endfeet around blood vessels (arrows in **o**). **p, q** Details of the caudal telencephalon showing the presence of small cells close to blood vessels (empty arrowheads) and endfeet positive for GFAP surrounding blood vessels (arrowheads in **p**), as well as the presence of numerous endfeet in the roof of the caudal ventricle (arrows in **q**). Scale bars 200 µm (**o**), 100 µm (**b, g, i, j, p**), 50 µM (**a, h, j′, m, n, q**), 20 µM (**c, d, e, f, k**), 10 µM (**g′, l**)

At S25, the telencephalic walls consist of a pseudostratified neuroepithelium that presents numerous cells that show weak GFAP immunolabeling in the cytoplasm. At this stage, a band of intense GFAP-immunoreactive processes is observed subpially (Fig. 4a).

At stage S29, GFAP immunoreactivity is present in numerous cells distributed from rostral to caudal regions of the telencephalic hemispheres (Figs. 2, 4b). GFAP immunoreactivity is more intense in the medial regions (Fig. 4c, d) than in lateral regions of the telencephalon (Fig. 4e). Numerous cells show GFAP immunoreactive processes running radially, with the proximal regions near the ventricular surface and the apical region enlarging subpially (resembling the radial glia of mammals). The shape of cells is ill-defined with this glial marker, and only in some cells the nucleus appears outlined by GFAP immunoreactivity. At these stages an intense immunoreactivity has also been detected in epithelial cells of the choroid plexus (Fig. 2f).

In the telencephalon of S30 and S31 embryos, the walls become thickened with the appearance of a thick mantle formed of neurons, whereas the ventricular zone is formed by one or two rows of radial glial cells that show GFAP immunoreactivity around the cell nucleus (Fig. 4g, g′). Numerous GFAP-immunoreactive radial processes originated from these cells are arranged as a palisade, crossing the mantle zone and extending to the pial surface (Fig. 2a′ at S31; Fig. 4g), where their hollow conical endfeet show an intense GFAP immunoreactivity (Fig. 4g). The thickness of the telencephalic wall and the length of radial glial processes vary among regions. In the rostral telencephalon, no differences in the intensity of immunoreactivity have been detected between medial and lateral territories. Caudally, a more intense immunoreactivity can be visualized in medial telencephalic territories than in lateral regions. Scarce GFAP-immunoreactive cell bodies are also observed alone or in small groups in the mantle zone. In the subpallium, GFAP-immunoreactive processes converge toward the midline in the medial ganglionic eminence homologue (Fig. 2b′, c′ at S31; Fig. 4h). Moreover, in this region, radial processes show a more complex, branched appearance in the marginal layer, which is in contrast with that observed in more immature pallial regions. Like in the previous embryonic stages, epithelial cells of the choroid plexus show intense GFAP immunoreactivity (asterisk in Fig. 4g). At these stages, GFAP-immunoreactive endfeet-like structures have also been observed around blood vessels.

In S32 embryos, the number of rows of GFAP-immunoreactive cells lining the lateral telencephalic ventricles increases and a few immunoreactive cells are observed in the mantle zone (Fig. 2a′–d′ at S32). In the dorsal telencephalic walls, faint GFAP-immunoreactive radial processes were appreciable in the rostral pallium. In the subpallium, numerous GFAP-immunoreactive processes converge toward the midline and show a more complex, branched appearance in the middle of this area, which also contains some interspersed intense GFAP-immunoreactive cells (Fig. 2a′, b′ at S32; Fig. 4i). More caudally, in the pallium, arched GFAP-immunoreactive processes form a curved palisade from the ventricular zone to the meningeal surface (Fig. 2c′ at S32; Fig. 4j) and numerous GFAP-immunoreactive cells are observed in the ventricular zone (Fig. 4j′). At this level, in the medial pallium, numerous GFAP-immunoreactive processes with an intensely stained endfeet are observed the pallial midline (Fig. 4k).

In lateral regions of the subpallium, no GFAP-immunoreactive processes are observed, although GFAP-positive subpial endfeet are present. In the caudal telencephalon, numerous GFAP-immunoreactive radial processes appear to course longitudinally owing to the tangential orientation of the section with regard to the dorsal pallium, although in the adjacent lateral pallium numerous radial processes are appreciable (Fig. 2d′ at S32). In the caudal subpallium only scarce and weakly GFAP-immunoreactive glial processes were observed. In the telencephalon of S32 embryos, GFAP-positive endfeet are also observed around blood vessels.

In posthatching juveniles, the thickness of the walls of the telencephalic hemispheres increases with respect to the embryonic period while that of the ventricular zone decreases. We have observed GFAP-immunoreactive ependymal cells (or tanycytes, Horstmann 1954) with a stained radial process lining the ventricle, their number being higher in the pallium than in the subpallium (Figs. 3a′–d′, 4l, m). Numerous GFAP-immunoreactive subpial endfeet, as well as perivascular endfeet surrounding blood vessels are observed in the pallium and subpallium (Fig. 4n, o). The dorsal and medial pallium showed numerous GFAP-immunoreactive processes, while in the presumptive ventral pallium and the subpallium such processes were barely stained (Fig. 4l). As in embryos, numerous GFAP-immunoreactive processes converge in the midline of the telencephalon and also toward the external sulcus between the presumptive ventral pallium and the olfactory bulbs (Fig. 3c′). In caudal telencephalon, a few GFAP-immunoreactive cells with round perikarya were scattered in the centromedial region of the dorsal pallium; these cells exhibit an immunoreactive process ending on blood vessels (Fig. 4p). Numerous GFAP-immunoreactive subpial endfeet are observed below the pial surface, and in the ventricular roof of the caudal telencephalic ventricle (Fig. 4q). In addition, “longitudinal” (rostrocaudal) GFAP-immunoreactive processes are also observed in the most caudal pallium, like in embryos (Fig. 3d′), and numerous GFAP-immunoreactive endfeet establish contact with blood vessels.

### Expression pattern of BLBP in the telencephalic hemispheres of embryos and juveniles

**Fig. 5:**
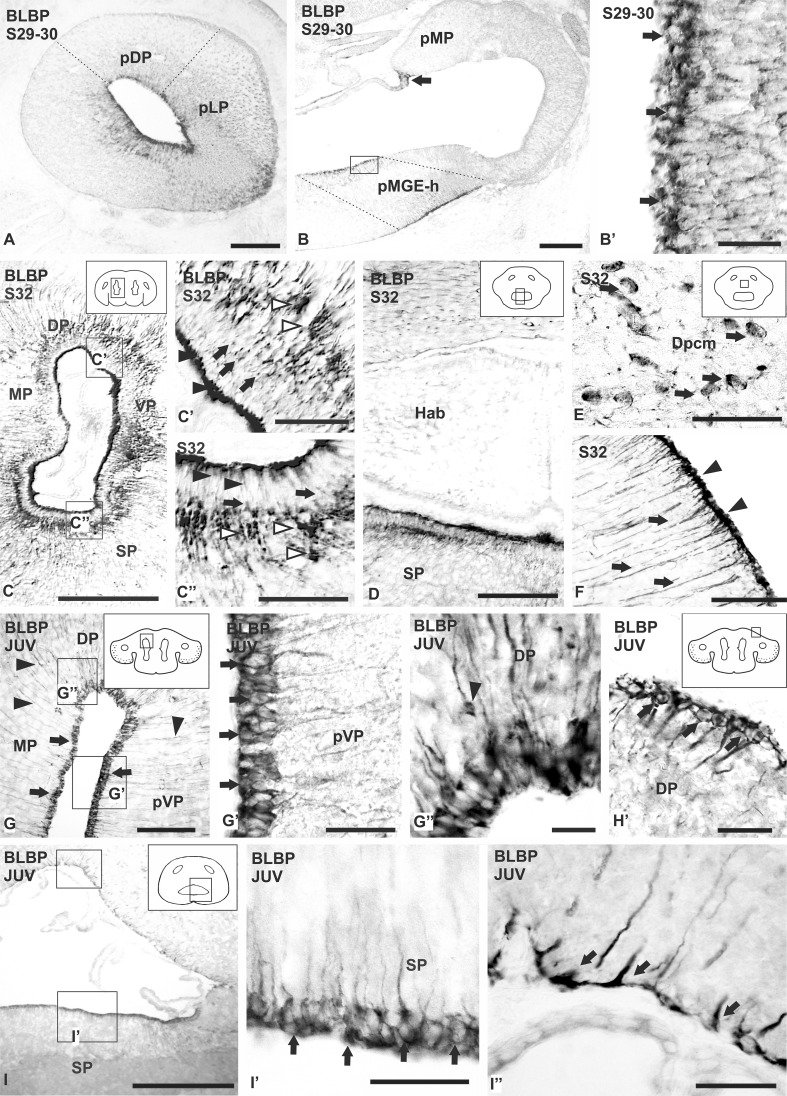
Photomicrographs of different sections of the telencephalon of *S. canicula* showing the expression pattern of BLBP during development (**a**–**f**) and juveniles (**g**–**i**′′). **a**–**b**′ Sagittal sections of the telencephalon of a S29–30 embryo showing BLBP immunoreactivity restricted to lateral portions of the pallium (**a**) and to medial portions of the subpallium (**b**). Note the intense immunolabeling in the choroid plexus (arrow). **b**′ Detail of the ventricular surface of the pMGE-h showing ventricular contacting positive cells (arrows). **c**–**f** Transverse sections of the telencephalon of a S32 showing BLBP immunolabeling. BLBP positive cells are mainly restricted to the ventricular zone (**c**), both in pallium (**c**′) and subpallium (**c**′′). Note that immunoreactivity is concentrated in the basal (arrows) and apical portion of the cells (arrowheads). Dilatations are pointed with empty arrowheads. **d** Photomicrograph of the caudal telencephalon showing intense immunolabeling in the ventricular zone of the subpallium. **e** Magnification showing BLBP positive cells located far away from the ventricular zone in the centromedial portion of the caudal dorsal pallium (arrows). **f** Transverse section showing the pial surface where radial processes (arrows) and endfeet structures (arrowheads) positive for BLBP are observed. **g**–**i**′′ Photomicrographs at different magnifications showing BLBP expression pattern in juveniles of *S. canicula*. **g** Transverse section of the pallium showing immunoreactivity for BLBP in ventricular cells (arrows) and in long radial prolongations (arrowheads). **g**′–**g**′′ Details of the ventral and dorsal pallial ventricle, respectively, showing BLBP positive cells in the ventricular zone (arrows) and a few in subventricular positions (arrowheads). **h** Detail of a group of BLBP positive cells at the pial surface of the pallium (arrows). **i** Panoramic view of the caudal telencephalon showing magnifications of BLBP positive cells in the subpallial ventricular zone (arrows in **i**′) and endfeet in the ventricular roof (arrows in **i**′′). Scale bars 200 µm (**a**–**d, g, i**), 100 µm (**c**′, **c′′, f**), 50 µm (**b**′, **e, g**′, **h**′, **i**′, **i′′**), 20 µm (**g**′′)

BLBP immunolabeling is detected in the telencephalon of *S. canicula* from S29 embryos onwards. At S29, BLBP immunoreactivity is restricted to dorsolateral regions of the pallium and the presumptive homolog of the medial ganglionic eminence (MGE-h) of the subpallium (Fig. 2a′′ at S29; Fig. 5a, b), where numerous ventricular cells and cells away the ventricular surface are observed. BLBP immunoreactivity is more intense in cells near the ventricle, which frequently show a bipolar morphology (Fig. 5b′). Numerous BLBP-immunoreactive subpial endfeet are also observed. In addition, strong BLBP immunoreactivity is detected in epithelial cells of the choroid plexus adjacent to the walls of telencephalic hemispheres. At the S31 stage, BLBP immunoreactivity is localized in the same regions of the telencephalon than in S29 and S30, but the amount of cells and fibers is higher and the intensity of staining was stronger. (Fig. 2a′′–c′′ of S31).

At the stage S32, strong BLBP immunoreactivity is observed in the whole ventricular border of the lateral ventricle at rostral and medial telencephalic levels (Fig. 2a′′–b′′ at S32). Immunoreactivity is observed in the apical region of cell bodies that line the ventricle and in radial processes that present dilatations away the ventricular zone, giving a dotted appearance to the paraventricular region. These dilatations are not uniformly distributed, being mostly located in dorsal and lateral pallial regions, as well as in the ventricular zone of the subpallium (Fig. 5c, c′, c′′). In the caudal subpallium, BLBP immunoreactivity is mostly observed in the ventricular zone of lateral subpallial regions (Fig. 2c′′ at S32; Fig. 5d). Groups of BLBP-positive cells are observed in the caudal part of the dorso-medial pallium (Fig. 2d′′ at S32). These round and large cells have an eccentric nucleus and some of them show a thick process (Fig. 5e). In the pallium and subpallium numerous BLBP-immunoreactive radial processes are observed emerging from the ventricular cells and coursing to the pial surface or converging in the telencephalic midline. Caudally, the number of immunoreactive processes coursing to the pial surface is higher in the pallium than in the subpallium (Fig. 2a′′–d′′ at S32). Numerous, strongly BLBP-immunoreactive subpial endfeet are observed in the telencephalon from rostral to caudal levels (Fig. 5f).

In juveniles, from rostral to caudal levels of the telencephalon, intensely BLBP-immunolabeled ependymocytes (or tanycytes) are observed along the surface of the lateral ventricles, which seem to be more numerous in the dorsal, lateral and ventral ventricular zones than in the medial ventricular zone (Figs. 3a′′–c′′, 5g). These cells show oval-shaped perikarya from which a basal BLBP-immunoreactive radial process extend, perpendicularly in some of them (Fig. 5g′–g′′). In addition, groups of a few BLBP-immunoreactive cells are observed in the pial surface of the dorsolateral telencephalic region (Fig. 3b′′). These cells show a rounded cell body with a BLBP-immunoreactive process oriented perpendicularly to the pial surface (Fig. 5h). BLBP immunoreactivity decreases in the caudal telencephalon. At this level, BLBP-expressing ependymal cells (tanycytes) are observed in the subpallial ventricle, but not in the choroid plexus (Figs. 3d′′, 5i, i′). Numerous BLBP-immunoreactive subpial endfeet are observed, and weakly BLBP-immunoreactive radial glial processes course from the ventricular zone to the pia forming subpial endfeet (Fig. 5i′′). Furthermore, BLBP-immunoreactive endfeet are observed on the surface of blood vessels.

### Expression pattern of GS in the telencephalic hemispheres of embryonic stages and juveniles

**Fig. 6:**
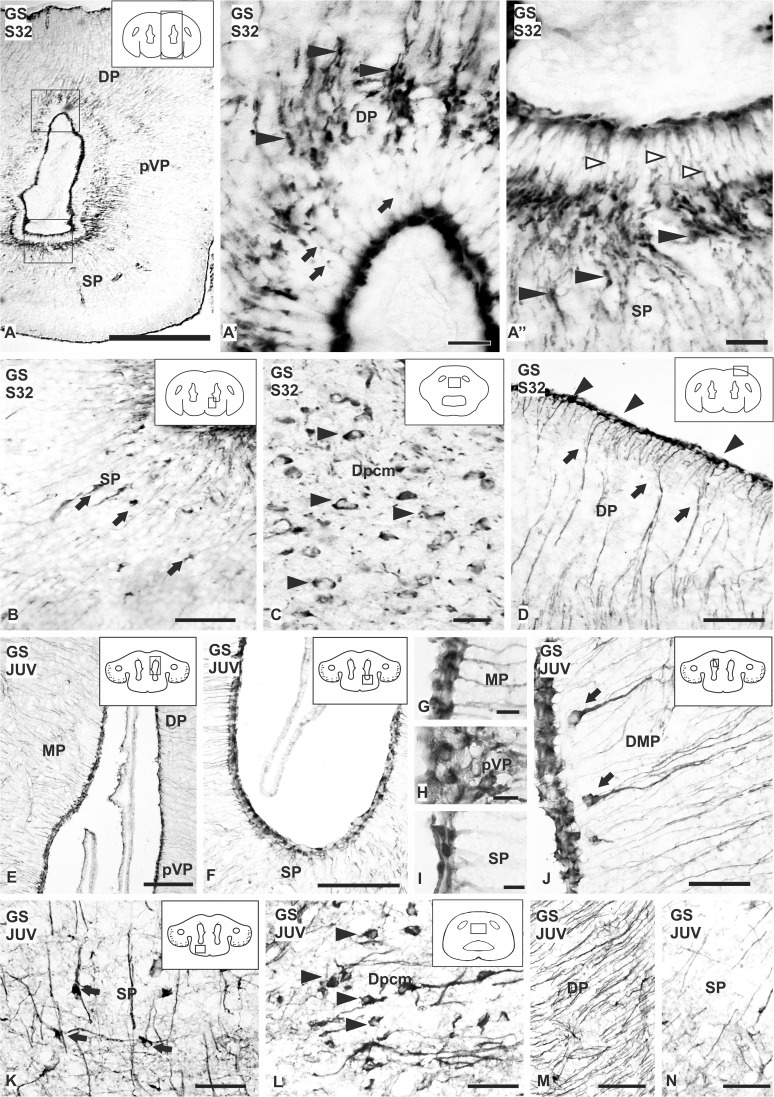
Photomicrographs of different sections of the telencephalon of *Scyliorhinus canicula* showing the expression pattern of GS during development (**a**–**d**) and juveniles (**e**–**n**). **a** Panoramic view of a transverse section of the telencephalon of a S32 of development showing GS immunolabeling. GS positive cells are mainly restricted to the ventricular zone, both in pallium (arrows) (**a**′) and subpallium (empty arrowheads) (**a**′′). Dilatations are pointed with arrowheads in **a**′–**a**′′. **b** Disperse GS positive cells are present in the subpallium near to the ventricular zone (arrows) and large cells are observed in the caudal dorsal pallium pars centromedialis (arrowheads) (**c**). **d** Photomicrograph showing radial processes (arrows) projecting to the pial surface, where they ramify and finish in endfeet (arrowheads). **e**–**n** Transverse sections of the telencephalon of catshark showing immunoreactivity for GS in the ventricular cells in pallium (**e**) and subpallium (**f**). **g**–**i** Details of the ventricular zone of the telencephalon at different levels showing GS positive cells. **j** Transverse section of the pallial ventricle showing paraventricular radial cells with a long basal prolongation (arrows). **k, l** Photomicrographs of disperse cells in the subpallium (arrows) and in the caudal telencephalon (arrowheads), respectively, immunoreactive for GS. **m, n** Transverse sections of the telencephalon showing differences in the amount of immunoreactive processes between pallium (**m**) and subpallium (**n**). Scale bars: 500 µm (**a**), 200 µm (**e, f**), 100 µm (**b**), 50 µm (**d, j**–**n**), 20 µm (**a′, a′′, c**), 10 µm (**g**–**i**)

No GS immunolabelling is detected in the telencephalon before S32. At this stage, intense GS immunoreactivity is mainly observed in the ventricular pole of glial cells, in dilatations along radial processes near the ventricular zone and in subpial endfeet. Other regions of the cell body and the radial processes appear faintly stained. The pattern of GS immunoreactivity in the ventricular zone of S32 embryos is similar to the pattern of BLBP immunoreactivity observed at the same stage (Fig. 2a′′′–d′′′ at S32). The GS-immunoreactive dilatations of radial processes are more numerous in the subpallium than in the pallium, where less immunoreactive cells are present; differences in the number of immunoreactive cells between the dorsal, medial and lateral ventricular zones can also be appreciated (Fig. 6a–a′′).

In pallium and subpallium of S32 embryos, some GS-immunoreactive cells are observed in adventricular regions (Fig. 2b′′′–d′′′ at S32; Fig. 6b, c). Numerous large and GS-immunoreactive cells are seen in the medial and dorsomedial pallium, these cells resembling those immunolabelled with BLBP (Fig. 6c); a few smaller GS-immunoreactive cells are observed in the medial subpallium near the ventricular zone (Fig. 6b). In the pallium, wavy GS-immunoreactive processes are observed coursing from the ventricle to the pial surface, where intensely immunoreactive endfeet are seen (Fig. 6d), whereas in the subpallium most of the positive processes are straight (Fig. 2c′′′ at S32). In transverse sections of the caudal telencephalon, radial processes appear to run longitudinally through the dorsal pallium (Fig. 2d′′′ at S32).

The expression pattern of GS in juveniles was similar to that observed in embryos (Fig. 3a′′′–d′′′). Numerous GS-immunoreactive ependymal cells (or tanycytes) are seen, and GS- positive cells are also observed away the ependymal region. There are no evident differences between rostral and caudal telencephalic regions, but immunoreactive ependymal cells are more numerous in the dorsal and lateral pallium than in the medial pallium or the subpallium (Fig. 6e–i). In rostral and middle levels of the pallium, GS-positive cells are observed near the ventricular zone. These GS-immunoreactive cells exhibit a round cell body and a radial process (Fig. 6j). GS-immunoreactive cells with bipolar morphology are seen in the subpallium away the ependyma (Figs. 3a′′′–d′′′, 6k). In the caudal dorsal pallium, GS-immunoreactive cells similar those observed in S32 are observed (Figs. 3d′′′, 6l). From the rostral to the caudal telencephalon, GS-immunoreactive radial processes are observed both in the pallium and the subpallium (Fig. 6m, n), but they are especially abundant in the dorsal pallial subdivision, where the immunoreactive processes show a parallel and wavy arrangement. In the caudal telencephalon, specifically in the dorsal centromedial pallium, processes appear cross-sectioned in transverse sections (Fig. 3a′′′–d′′′).

### Double immunofluorescence of GFAP/GS and BLBP/GS

The similar pattern of GFAP, BLBP and GS immunoreactivities observed in the ventricular zone in latter stages of development led us to explore the possibility that the three glial markers were labelling the same glial populations. For this goal, double immunofluorescence combinations of GS and GFAP or BLBP were carried out and the cells of the ventricular zone have been analysed in detail for the presence of these markers.

The ventricular zone of the telencephalic regions exhibits different degrees of thickness from S32 embryos onwards. The ventricular zone of pallium is wider than the subpallial one. Meanwhile, the subpallial ventricular zone seems to be homogenous in thickness, in contrast to the pallium, where differences can be appreciated between the different pallial territories. The ventricular zone of the dorsal and medial pallium is clearly thinner than the ventricular zone of the presumptive ventral pallium. Moreover, the ventricular zone of the pallial/subpallial border seems to be substantially thicker than the adjacent ventricular zones.

**Fig. 7:**
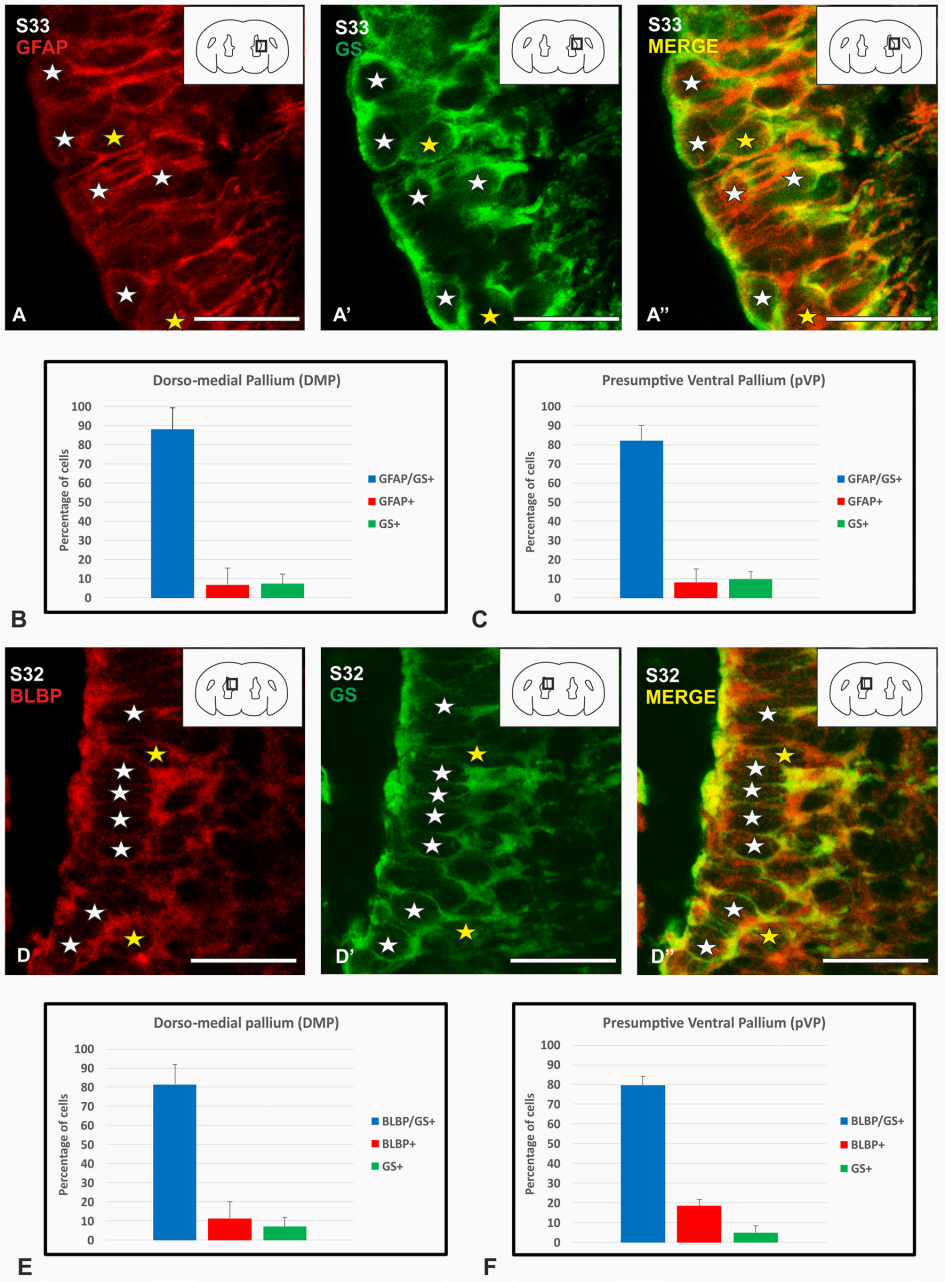
Photomicrographs of the telencephalic ventricle during development showing double immunolabeling for GFAP and GS (**a**–**a**′′), BLBP and GS (**d**–**d**′′) and cell counting (**b, c, e, f**). **a**–**a**′′ Details of the ventricular zone of a S33 of development showing double-immunolabelled cells (white stars) and a few cells where both glial markers do not colocalize (yellow stars). **b, c** Bar representations of percentage of GFAP/GS single and double-labelled cells in the dorso-medial pallium and presumptive ventral pallium, respectively. **d**–**d**′′ Details of the ventricular zone of a late S32 of development showing double-immunolabelled cells (white stars) and a few cells where both glial markers do not colocalize (yellow stars). **e**–**f** Bar representations of percentage of GFAP/GS single and double-labelled cells in the dorso-medial pallium and presumptive ventral pallium, respectively. Scale bars 25 µm

Double immunofluorescence against GFAP/GS was performed in the S32/33 stage. Results of this GFAP/GS double labelling show co-expression of both markers in most glial cells bordering the telencephalic ventricles. Numerous GFAP/GS-positive cells are observed in the dorsal, medial and presumptive ventral pallial ventricular zone (white stars Fig. 7a, a′, a′′), and in the subpallial ventricular zone. Although cells expressing both markers represent the majority of cells, a few cells express only one glial marker (yellow stars in Fig. 7a, a′, a′′). Cell counting approach in the pallium revealed that around 84% of cells where double-labelled, while the rest expressed only one glial marker (see graphics in Fig. 7b, c). Cells expressing GFAP or GS only represent similar percentages (around 8%). In the subpallium, all cells coexpress both markers. For an integrated graphic of all regions see Suppl. Fig. 1c.

Double immunofluorescence against BLBP/GS was performed in the S32/33 stage. Similarly to GFAP/GS double immunofluorescence, many double-labelled cells can be observed in different regions of the pallial ventricular zone (white stars in Figs. 7d–d′′). However, overall co-expression of both glial markers is observed in the subpallium. Cell counting in the pallium have shown that around 80% of cells where double-labelled cells, while the rest expressed or BLBP or GS alone (see graphics in Fig. 7e, f). In contrast to GFAP/GS, the percentage of cells expressing BLBP or GS alone shows differences between different portions of the pallium. In the subpallium all cells coexpress both markers. For an integrated graphic of all regions see Suppl. Fig. 1D.

### Double immunofluorescence to PCNA and GFAP or BLBP

**Fig. 8:**
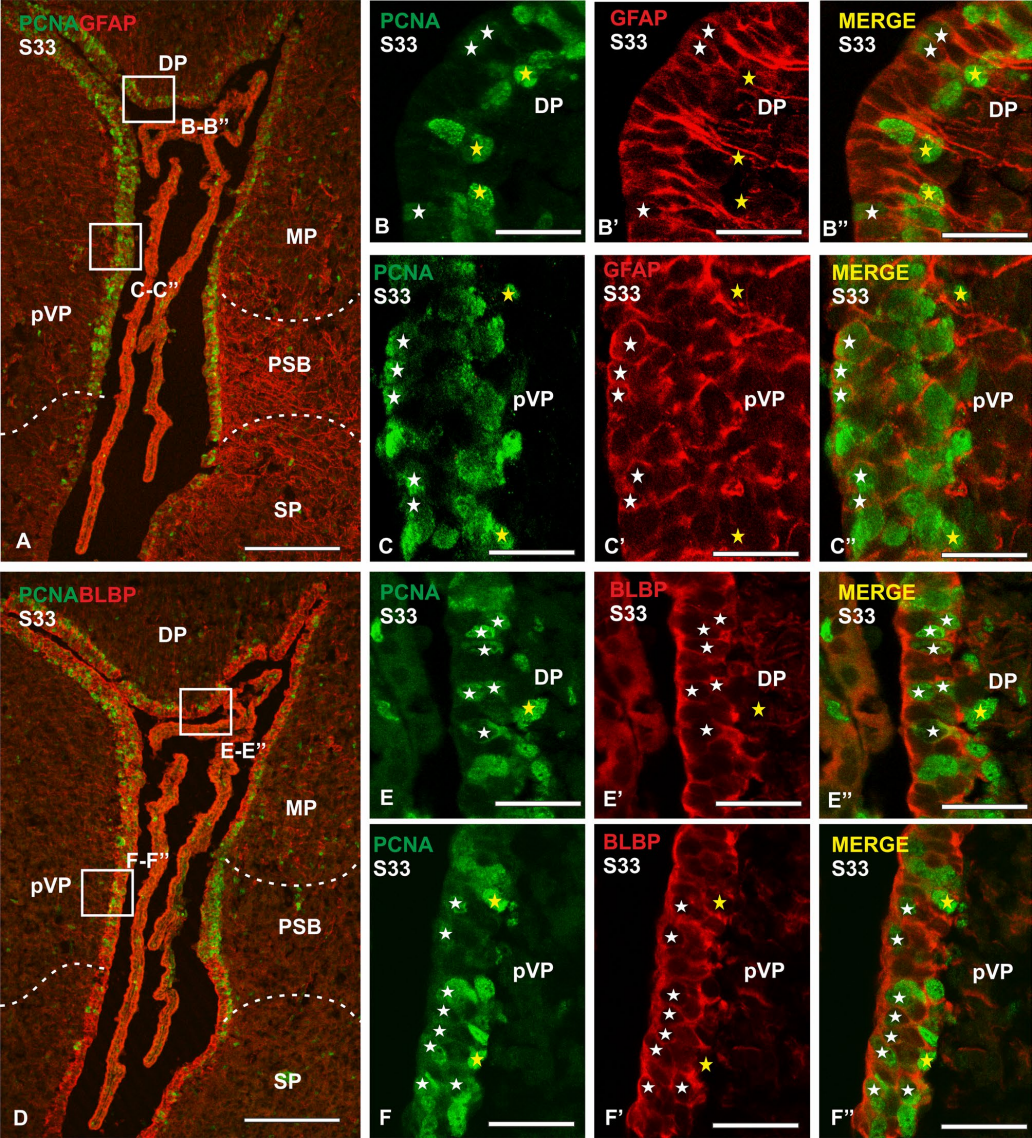
Photomicrographs of different sections of the telencephalon of *S. canicula* in S33 embryos showing double immunofluorescence for PCNA and GFAP or BLBP. **a** Panoramic view of the telencephalic ventricle showing double immunofluorescence for PCNA and GFAP. **b**–**c**′′ Details of the ventricular zone of dorsal pallium and presumptive ventral pallium showing numerous double-labelled cells (white stars) and some BLBP− PCNA+ cells (yellow stars). **d** Panoramic view of the telencephalic ventricle showing double immunofluorescence for PCNA and BLBP. **e**–**f**′′ Details of the ventricular zone of dorsal pallium and presumptive ventral pallium showing numerous double-labelled cells (white stars) and some BLBP− PCNA+ cells (yellow stars). In higher magnification details the ventricle is on the left side of the photomicrographs. Scale bars 200 µm (**a, d**), 25 µm (**b**–**b′′, c**–**c′′, e**–**e′′, f**–**f′′**)

At later embryonic stages, PCNA immunofluorescence shows numerous cells with PCNA-positive nuclei lining the ventricles. These proliferating cells are widely distributed along the pallial subdivisions, in contrast with the subpallium, where few PCNA-positive cells are appreciable. PCNA-immunoreactive cells are numerous in rostral levels of the telencephalon and its number decreases toward caudal levels. In middle sections of the telencephalon, where the pallium can be subdivided into dorsal, medial and presumptive ventral pallium, differences among these subdivisions are found as regards PCNA immunoreactivity. The dorsal and medial ventricular zones show scarcer PCNA-immunoreactive cells compared to the presumptive ventral pallial ventricular zone and the pallial–subpallial boundary. Faint immunolabelled cells are observed close to the ventricle, while PCNA-immunoreactive nuclei more intensely stained are located in the basal portions of the ventricular zone. For panoramic views see Fig. 8a or d. On the basis of this distribution pattern we have analysed the co-expression of PCNA and the RGCs markers GFAP and BLBP in S33 and S34 of development (final stages before hatching).

At S33 of development, double immunofluorescence against GFAP and PCNA reveals wide colocalization of both markers in cells of the telencephalic ependyma, especially in the pallial subdivision (Fig. 8a). In the dorsal (Fig. 8b–b′′) and presumptive ventral pallium (Fig. 8c–c′′) three populations of cells are distinguishable: GFAP +/PCNA+ cells (Fig. 6b–c′′), GFAP+/PCNA− cells and GFAP-/PCNA+ cells. The most abundant cell population is that consisting of GFAP+/PCNA+ cells. Interestingly, most of these cells show fainter PCNA immunoreactivity than those that do not co-express GFAP. The immunoreactivity against GFAP/PCNA in the ventricular zone of the medial pallium exhibits the same distribution than in other pallial subdivisions. Numerous double labelled cells are also detected along the pallial–subpallial boundary, where the thick ventricular zone shows a high number of proliferating cells.

Double immunofluorescence against BLBP and PCNA in S33 reveals wide codistribution in the telencephalic ventricular zone, with a high level of co-expression of both factors in the pallial subdivision (Fig. 8d). This distribution is similar to that of GFAP/PCNA double immunofluorescent cells. In the dorsal (Fig. 8e, e′, e′′) and ventral (Fig. 8e, e′, e′′) pallium, three different populations of cells are distinguishable: BLBP+/PCNA+ cells, BLBP+/PCNA− cells and BLBP−/PCNA+ cells. Numerous PCNA immunoreactive cells co-expressed BLBP but, as for GFAP and PCNA immunofluorescence, double labelled cells use to present fainter PCNA-immunoreactive nuclei compared to cells that do not co-express BLBP. Abundant double labelled cells are also detected in the boundary between the medial pallium and the subpallium. The expression of BLBP and PCNA in the ventricular zone of the medial pallium shows the same pattern than in other pallial subdivisions.

In addition, numerous scattered cells showing PCNA-positive nuclei are distributed away from the ventricular zone. A few of these cells co-express GFAP and show a different morphology from that of the PCNA-positive cells of the ventricular zone. Many BLBP-positive cells located away the ventricular regions also showed PCNA immunoreactivity.

**Fig. 9:**
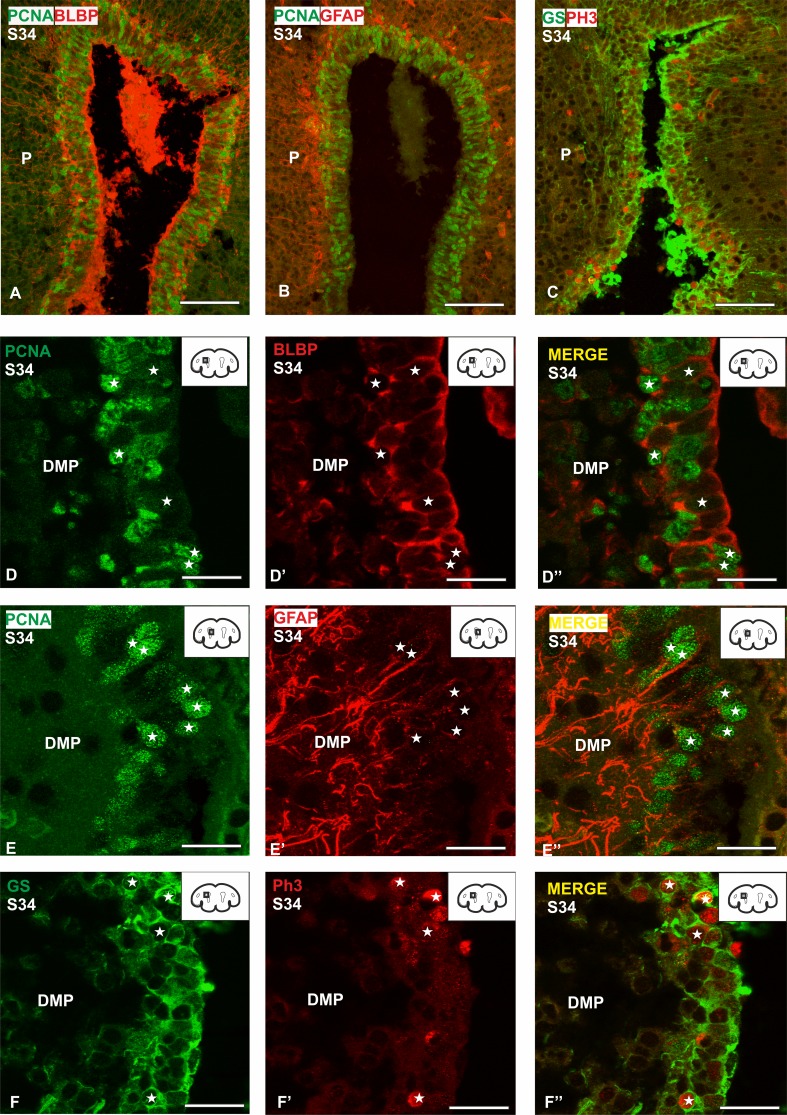
Transverse sections of the pallium of a S34 of development (prehatching) at different magnifications showing double immunofluorescence for the three glial markers BLBP, GFAP, GS and the proliferating markers PCNA, PH3. **a** Panoramic view of the telencephalic ventricle showing double immunofluorescence for BLBP and PCNA. **b** Panoramic view of the telencephalic ventricle showing double immunofluorescence for GFAP and PCNA. **c** Panoramic view of the telencephalic ventricle showing double immunofluorescence for GS and PH3. **d**–**d**′′ Details of the ventricular zone of a S34 of development showing double-immunolabeled cells for BLBP and PCNA (white stars). **e**–**e**′′ Details of the ventricular zone of a S34 of development showing double-immunolabeled cells for GFAP and PCNA (white stars). **f**–**f**′′ Details of the ventricular zone of a S34 of development showing double-immunolabeled cells for GS and PH3 (white stars). In higher magnification details the ventricle is on the right side of the photomicrographs. Scale bars: 100 µm (**a**–**c**) 25 µm (**d**–**f**′′)

Double immunofluorescence against BLBP/PCNA and GFAP/PCNA has also been performed in S34 embryos (Fig. 9a, b). Besides, double immunofluorescence between GS and PH3 has been performed (Fig. 9c). As in S33 embryos, both BLBP/PCNA and GFAP/PCNA immunofluorescence allows to observe numerous double labelled cells in the ventricular zone of the telencephalon (Fig. 9d–e′′). In addition, double immunofluorescence GS/PH3 evidences many double-positive cells (not as much as PCNA), in the ventricular zone of the pallium (Fig. 9f–f′′). In this case, no differences between the telencephalic subdivisions have been appreciated.

## Discussion

The present work represents the first study of the glial cytoarchitecture during the embryonic development of a cartilaginous fish using markers of radial glia and cell proliferation. We analysed the expression of GFAP, BLBP, GS, and PCNA by means of immunohistochemistry in the developing telencephalon of *S. canicula* in S25–S34 embryos, as well as in posthatching (early) juveniles. On the basis of major morphological characteristics, Ballard et al. (1993) established 3 periods during the development of *S. canicula*: (1) early development, from the day of fertilization until S26 of development; (2) intermediate development, from S27 to S31; (3) and late development, from S32 to hatching. More recently, equivalences among brain developmental stages of *S. canicula* and mouse, birds and zebrafish have also been established (Rodríguez-Moldes et al. 2017). From the day of fertilization until hatching, the catshark embryogenesis takes around 6 months (depending on water temperature), which is a lengthy period compared to the 72-h of zebrafish embryogenesis. Therefore, sharks provide a good time window for studying certain developmental processes that might be ignored by a rapid development (Quintana-Urzainqui et al. 2015; Rodríguez-Moldes et al. 2017). In addition, sharks exhibit an evaginated telencephalon, instead of an everted telencephalon as in teleost fishes, which ease comparisons between developmental periods and developmental events in sharks and other tetrapods.

We show that the expression pattern of the three glial markers studied follows a sequential expression pattern along development: early (GFAP), intermediate (GFAP and BLBP) and late (GFAP, BLBP and GS). We found that these markers mainly label RGCs in the ventricular zone of the developing telencephalon and that their expression remains in juveniles. In addition, some non-ventricular positive cells were found during development (BLBP- and GS-immunoreactive cells) and in juveniles (GS-inmunoreactive cells) which do not have morphology of RGCs. Ventricular zone RGCs usually co-express all glial markers studied in late development, but exhibit some degree of heterogeneity in the dorsal telencephalon. In addition, double immunofluorescence experiments have shown a high degree of colocalization between the proliferation marker (PCNA) and glial markers such as GFAP or BLBP. The comparative aspects across vertebrates and the developmental implications regarding the appearance of these cells will be discussed below.

### GFAP, BLBP and GS are expressed in cells with morphology of RGCs during embryonic development and in postnatal individuals

In the telencephalic hemispheres of the lesser spotted dogfish, GFAP immunoreactivity is detected in embryos from early developmental stages (S25) until the end of embryonic period (S32–S34), as well as in posthatching juveniles. In mouse embryos, GFAP mRNA was first detected in the germinal zones of the forebrain on E15 and it increases on E17 (Fox et al. 2004), which correspond to late embryonic stages (Rodríguez-Moldes et al. 2017). Mamber et al. (2012) showed that GFAPδ transcripts are expressed from E12 in radial glia, but GFAP-immunoreactive structures are only detected from E18 onwards. RGCs expressing GFAP were also detected in the forebrain of late embryonic stages of *Xenopus laevis* (S36) (Messenger and Warner 1989). Studies in zebrafish embryos have shown that the expression of GFAP in the central nervous system (CNS) begin at the time of formation of the first somite (Thisse et al. 2001) but, contrary to that observed in *S. canicula*, mouse and *Xenopus* its expression decreases during the embryonic period (Marcus and Easter 1995). The early developmental expression of GFAP in chondrichthyes and teleosts contrasts with its later expression in mammals. In mammals, the establishment of the RGCs scaffold during forebrain development necessary for neuronal migration occurs previously to the neurogenic period (Martynoga et al. 2012). Quintana-Urzainqui et al. (2015) established that neurogenesis and neuronal migration in the catshark telencephalon mainly occurs during the intermediate developmental period (S27–S31). Like in mammals, GFAP expression in the telencephalon of the catshark occurs before neurogenesis; therefore, the presence and distribution pattern of GFAP-positive radial processes in the telencephalic hemispheres at these stages indicate that they could act as guide-posts during neuronal migration, as do RGCs in the mammalian cortex (Rakic 1972). Regarding the distribution of GFAP within glial cells, in early embryos GFAP is mainly located in subpial endfeet. As embryonic development progresses, GFAP is observed in cell bodies lining the ventricle and its radial processes and subpial endfeet. These GFAP-immunoreactive cells morphologically resemble glial cells described in the developing cerebral cortex of mammals (for review see: Alvarez-Buylla and Kriegstein 2013). The distribution pattern of GFAP in catshark embryos also resembles that shown in studies of amphibians (Messenger and Warner 1989) and teleosts (Marcus and Easter 1995; Arochena et al. 2004). A similar distribution pattern is found in early juveniles, though GFAP expression decreases with respect to that observed in embryos: the thickness of the ependyma decreases with respect to the ventricular zone of embryonic stages, and less GFAP immunoreactive ependymocytes or tanycytes are visualized in contact with the ventricle. In any case, radial glial processes and subpial endfeet still contain GFAP.

The distribution pattern of GFAP observed in the telencephalon of posthatching juveniles is in agreement with the GFAP distribution pattern reported in the adult brain of reptiles (Lazzari and Franceschini 2006), amphibians (Kirkham et al. 2014) and diverse bony fishes (carp: Kálmán 1998; trout: Díaz-Regueira and Anadón 1998; grey mullet: Arochena et al. 2004; zebrafish: Grupp et al. 2010; Than-Trong and Bally-Cuif 2015; african lungfish: Lazzari and Franceschini 2004) and chondrichthyes (sharks, rays and skates: Wasowicz et al. 1999; Kálmán and Gould 2001; Ari and Kálmán 2008b; and chimaeras: Ari and Kálmán 2008a), in which it has been shown that ependymal and radial glial cells are the predominant astroglial cell type. However, GFAP positive cells are found in the ventral telencephalon of catshark posthatching juveniles, which is not the case in teleost fish, such as zebrafish (Ganz et al. 2010; März et al. 2010), where GFAP expression is essentially absent. Zebrafish and catshark show many differences in their embryonic development; zebrafish is fast developmental species, while catshark is slow developmental species, and the telencephalon morphogenesis process is also different between both species. In addition, galeomorph sharks have relatively large brains, similar in size to those of the mammal and bird brains with similar body weights (Northcutt 1981) and its size brain increases from posthaching period until adulthood. During mammalian embryonic development the length of radial glia processes has been related with the radial growing of the neural tube, in addition, the number of radial glial cells determine the size of a brain region, and at the same time radial glia cells act as guides in the neuronal migration process (for review see Götz 2013). The presence of GFAP in the ventral telencephalon of catshark may be related with the continuous growth of the brain.

In the adult brain of skates, GFAP-positive cells with stellate morphology typical of astrocytes were also found (Kálmán and Gould 2001). Although we have not observed astrocyte-like cells in the telencephalic hemispheres of juveniles, GFAP-immunoreactive cells with astrocytic morphology can be visualized in other brain areas.

BLBP-immunoreactivity in RGCs of the catshark telencephalic hemispheres is detected after GFAP-immunoreactivity, around S28 (present results). At this developmental stage BLBP-immunoreactivity is weak and restricted to ventricular cells, but as development proceeds (S31–S32) its expression increases considerably and can also be observed in cells away of the ventricular zone (present results). In mammals, BLBP is a marker of radial glia and its expression is first visualized in RGCs present in the caudolateral regions of the forebrain on E12.5 and, by E14.5–E16.5, BLBP expression expands to medial regions of the forebrain (Anthony and Heintz 2008). Rodríguez-Moldes et al. (2017) established equivalences between catshark S27–S31 and mouse E10.5–14.5 stages, which suggests striking similarities in the temporal expression of BLBP between catshark and mouse. In *Xenopus* BLBP-positive cells line the telencephalic ventricles at stages 37/38 (Moreno and González 2017). These *Xenopus* stages belong to a late embryonic period; however, as they show active neurogenesis it can be comparable to the catshark intermediate period. In the zebrafish forebrain, BLBP positive cells were described lining the ventricles in adults and 21 dpf juveniles (Grupp et al. 2010). We are not aware of studies of BLBP expression during zebrafish embryogenesis, precluding further comparisons.

A subpial layer of BLBP immunoreactive cells has been detected from S32–33 onwards. GFAP-immunoreactive subpial astrocytes have been described in the telencephalon of adult rays (Kálmán and Gould 2001), however, the morphology of the catshark BLBP-positive subpial cells resembles more to RGCs than to ordinary astrocytes, suggesting interspecific differences. In contrast with other vertebrates, the blood–brain barrier in cartilaginous fishes is not formed by endothelial cells but is formed by glial cell processes (Bundgaard and Cserr 1981). Whether the submeningeal glial cells observed in cartilaginous fishes contribute to form the blood–brain barrier need to be further investigated.

In catshark embryos, numerous BLBP-positive cells line the telencephalic ventricles and their immunoreactive radial processes span the telencephalic walls from the ventricular zone to the pia. Scattered BLBP-immunoreactive cells outside the ventricular zone were also observed. However, in juveniles BLBP expression was restricted to ventricular glial cells and their subpial endfeet (present results). In mammals, BLBP is transiently expressed in the RGCs in the embryonic ventricular zone. It has been proposed that BLBP is involved in the establishment of the radial processes of RGCs in the developing brain (Feng et al. 1994). Studies performed in zebrafish (März et al. 2010; Diotel et al. 2016) and *Xenopus* (D’Amico et al. 2011) have reported the presence of BLBP in RGCs of juvenile and adult brains, with a distribution pattern similar to that observed in the telencephalon of juvenile catshark. Likewise, BLBP expression in the catshark telencephalon might be related with the formation of the radial glia scaffold that persists in juveniles.

GS expression in the telencephalon of catshark appears in the later embryonic period previously to hatching, and the distribution pattern evidenced during embryonic development is similar to that of BLBP. In mammals GS is present in astrocytes and RGCs; in rat embryos, GS immunoreactivity was first observed in ventral ependymocytes of caudal neural tube at embryonic day 14 (E14) and radial glia shows GS immunoreactivity at E16 (Akimoto et al. 1993). As in the rodent brain, in the catshark telencephalon GS is expressed in the later period of embryonic development. However, in teleosts and reptiles GS occurs earlier in development (Romero-Alemán et al. 2003; Grupp et al. 2010). The enzyme GS has a critical role in the metabolism of neurotransmitters released by neurons such as glutamate, which is uptaken by glia and converted by GS to glutamine to prevent neurotoxicity (Norenberg 1979). GS has been reported as a marker of mature glial cells in the retina of this catshark species (Bejarano-Escobar et al. 2012; Sánchez-Farías and Candal 2016). In juveniles GS was detected in ependymal cells (or tanycytes), which is in agreement with results observed in the adult human and mouse brain GS (Norenberg 1979; Norenberg and Martinez-Hernandez 1979; Bernstein et al. 2014); moreover, GS positive cells with morphology of RGCs were also found in the adult brain of amphibians (Kirkham et al. 2014) and fishes (teleosts: Grupp et al. 2010; Than-Trong and Bally-Cuif 2015; chondrichthyes: Ari and Kálmán 2008a, b).

We can conclude that GFAP, BLBP and GS label cells with radial glia morphology during different developmental periods in the telencephalon of catshark. The onset expression timing of the three glial markers during catshark embryonic development shows many similarities with mammals. Present results reinforce the hypothesis that the basic developmental processes in this species (main molecular events, main morphogenetic processes) are quite similar to those of mammals (see also Rodríguez-Moldes et al. 2017 and references therein). However, in the catshark telencephalon RGCs are maintained after hatching, as in other species of fishes, which has been related with the high neurogenic potential of the glia in adult fishes (reviewed by Than-Trong and Bally-Cuif 2015). Present results also indicate that the lesser spotted dogfish is an excellent model to study the development of the glial system in an evo-devo context.

### BLBP and GS adventricular positive cells may belong to the oligodendroglial lineage

In embryonic and juvenile stages of catshark, scattered GS-positive cells are observed in the mantle zone of the medial and the caudal dorsal pallium (pars centromedialis). These cells also express BLBP in embryos, but not in juveniles (present results). They are characterized by their large size, the eccentric nucleus and a thick process, features that resemble to those of oligodendrocytes. Del Río Hortega (1928) made the first description of oligodendrocytes and classified them into four types attending to their size, shape, processes or even their interaction with axons (reviewed by Pérez-Cerdá et al. 2015). We found that GS-positive cells of the caudal dorsal pallium in juvenile fit well with type-3 oligodendrocytes, which are described by Del Rio Hortega as cells with a “bulky cell body” with one to four processes. In reptiles, it was shown that most of the cells positive for S-100 (also used as a marker of oligodendrocytes) also express GS during the embryonic period (Romero-Alemán et al. 2003). Interestingly, in adult humans and rodents, GS is expressed by subpopulations of oligodendrocytes (Bernstein et al. 2014). Ultrastructural studies focused on the mesencephalon of a lizard indicated that GS-positive cells may be oligodendrocytes (Monzón-Mayor et al. 1998). Oligodendrocytes with different size and shape were found in the brain of rainbow trout (Pérez et al. 1996) using NADPH diaphorase enzymohistochemistry, and these are morphologically similar to the GS-positive mantle cells of the catshark telencephalon. Differences in the size of oligodendrocytes has been related with the size of axons they ensheath (Díaz-Regueira and Anadón 1998). Fibres in the catshark telencephalon are mostly unmyelinated, except in the compact bundles that run through the medial pallium and the centromedial dorsal pallium (Smeets et al. 1983). Curiously, we have also observed similar cells positive for GS in the rhombencephalon of this specie associated with myelinated tracts of large diameter such as the medial longitudinal fascicle (results not shown). GS immunohistochemistry also revealed adventricularly disperse cells in the subpallium with a different morphology. These bipolar cells are orientated radially and horizontally in the subpallium and exhibit thin processes. They might correspond to either radial glial cells that have lost contact with the ventricular zone or to type-4 oligodendrocytes (or Schwannoid cells) of the classification of Del Rio Hortega, which use to show bipolar processes adhered to thick axons (for review see: Pérez-Cerdá et al. 2015). Numerous thick axons, some of them myelinated, run in the basal forebrain bundles through the catshark subpallium, including the area superficialis basalis (Smeets et al. 1983).

Since we have detected GS-positive cell type that morphologically resemble oligodendrocytes in regions that contain myelinated fibres we have carried out double immunofluorescence techniques using antibodies against GS and chondroitin sulfate proteoglycan NG2 (a marker of oligodendrocyte precursor cells, OPCs). Though GS-positive cell populations do not express NG2 (data not shown), we cannot exclude the possibility that these cells belong to the oligodendroglial lineage. Further investigations using markers of late progenitor pro-oligodendrocytes and mature oligodendrocytes such as O4-antigen and myelin proteins (Ono and Ikenaka 2013) might shed light about the nature of these glial cells.

### Subsets of proliferating RGC are present in the catshark embryonic telencephalic ventricular zone

In mammals, the development of the forebrain involves multiple types of progenitor cells, including NECs, RGCs, different pools of intermediate progenitor cells and basal radial glia cells. Apical progenitor cells (or RGCs) are primary progenitor cells generated from NECs; they undergo self-renewing division in the ventricular surface, producing neurons and generating different pools of intermediate progenitors that themselves divide and give rise to differentiated neurons or different types of glial cells (astrocytes, oligodendrocytes and ependymal cells) at sequential stages in development. Although by the end of development most of the RGCs transform into astrocytes, in some brain areas RGCs persist after birth and function as primary progenitor cell (Götz et al. 1998; Marshall et al. 2003; Spassky et al. 2005; Kriegstein and Alvarez-Buylla 2009; Taverna et al. 2014; Beattie and Hippenmeyer 2017).

In mammals, the transition from NECs to apical RGCs involves the expression of several glial markers, including BLBP; several studies have shown that activation of the BLBP promoter in NECs promotes radial glia differentiation (for review see: Pinto and Götz 2007). In rodents most of the precursor cells in the embryonic ventricular zone are RGCs (Noctor et al. 2002). We have detected that most of the ventricular cells express BLBP in S29–S30 catshark embryos, suggesting that these are RGCs progenitors and that the transition from NECs (BLBP-negative) cells to RGCs occurs before these stages. Other feature of the apical RGCs of the mammalian dorsal telencephalon is the expression of the paired-box transcription factor 6 (Pax6) (Englund et al. 2005). Previous studies of our group have found expression of Pax6 in the dorsal telencephalon of catshark at S29 (results not shown). This data suggests that most of the BLBP-positive cells in the dorsal telencephalon also co-express Pax6.

On the other hand, our results indicate that the surface of the catshark telencephalic ventricles in later embryonic stages consists of subsets of RGCs; double immunofluorescence against GFAP, BLBP and GS reveals the presence of subpopulations of RGCs in the ventricular surface (see Fig. 7 and Suppl. Figure 1). Due to the incompatibility among the antibodies used, double immunofluorescence between GFAP and BLBP was not carried out. However, according to the distribution pattern we observed, a high degree of colocalization between GFAP and BLBP is expected. A molecular heterogeneity of RGCs is present in the developing telencephalon of mammals, with subsets of radial glial cells differing in the expression of the GLutamate ASpartate Transporter (GLAST), BLBP and the Radial Glial cell marker 2 (RC2), and whose proportions change throughout the neurogenic period (Hartfuss et al. 2001). In catshark embryos, we have not appreciated regional differences in the distribution of the subsets of radial glial although variations in thickness of the ventricular zone were observed.

Most of the RGCs expressing GFAP and BLBP are PCNA-positive, which indicates that they are proliferative (present results). Previous studies in this catshark have reported a high proliferative potential in S32–S34 embryos (Quintana-Urzainqui et al. 2015). Using fluorescently-tagged endogenous proliferating cell nuclear antigen (PCNA), Zerjatke et al. (2017) showed that differences in the intensity of the PCNA immunolabelling reflect different phases of the cell cycle. Based on this fact, GFAP- and BLBP-positive cells of the catshark telencephalon with intense PCNA are probably in S phase, while nuclei with a weak PCNA immunolabelling probably indicate cells that are in other phases of the cell cycle. In the mammalian telencephalon, nuclei undergoing the S phase form several layers at the basal surface of the ventricular zone. On the other hand, nuclei in M phase are close to the ventricle, and nuclei in G1 and G2 phases are in the mid region. This process is known as “interkinetic nuclear migration” (for review see: Kriegstein and Alvarez-Buylla 2009). Such zonation of the ventricular zone can be observed on the basis of the intensity of PCNA immunoreactivity in the telencephalon of catshark.

On the other hand, the number of proliferating ventricular cells seems higher in the pallium than in the subpallium. Differences in the proliferative potential between the dorsal and ventral telencephalon were also described in rodent embryos (Malatesta et al. 2003). Our results are in agreement with studies in other vertebrates showing proliferative RGCs. In mouse embryos, most of the proliferating ventricular cells have morphological and molecular characteristics of radial glia and express BLBP (Noctor et al. 2002; Anthony et al. 2004). Proliferating BLBP-positive cells are also present in the ventricular zone of *Xenopus* embryos (Moreno and González 2017) and teleosts (Grupp et al. 2010). Furthermore, in the developing dorsal telencephalon of reptiles ventricular radial glial cells undergo cell divisions (Clinton et al. 2014). Like in mammals (Hartfuss et al. 2001; Noctor et al. 2002), RGCs represent the majority of precursor cells in the catshark telencephalon. Our results also indicate that different cycling cells are present in the ventricular zone. Double GFAP/ GS and BLBP/GS immunolabelling clearly showed the presence of different RGCs subpopulations, and double immunofluorescence for PCNA and GFAP or BLBP revealed some proliferating cells that do not exhibit glial cell markers. Curiously, nuclei of most of these proliferating cells used to be strongly PCNA-immunoreactive, which means that cells are in S phase, and in consequence committed to undergo mitosis. Despite the actual nature of these cells is not known, they might correspond with some kind of intermediate progenitor cells.

Fate mapping experiments using Cre recombinase driven by RGCs promoters BLBP or GFAP in mammals indicate that most neurons throughout telencephalon originate from RGCs that express GFAP and BLBP and, interestingly, GFAP-positive cells also produce oligodendrocytes and astrocytes (Malatesta et al. 2003; Casper and McCarthy 2006; Anthony and Heintz 2008; Farkas and Huttner 2008; Martynoga et al. 2012). Besides, in rodents RGCs give rise to most of the ependymal cells between E14 and E16, and these cells complete their maturation after birth (Spassky et al. 2005). If these results were extensive to basal vertebrates such as sharks, it would suggest that BLBP/GFAP-positive proliferating cells may generate both neurons and different types of glial cell at the end of the embryonic period. At this point, further investigations are needed to elucidate the identity of the progeny originated by the proliferating RGCs present in the catshark telencephalon.

## Conclusion

This is the first study during embryogenesis focussed on the development of RGCs using different glial markers in the telencephalon of the catshark. Radial glial markers such as GFAP, BLBP and GS are sequentially expressed along the development and its expression/distribution becomes overlapping and persistent in the postnatal telencephalon, evidencing a wide and organized RGCs pattern. We found that RGCs exhibit certain degree of molecular heterogeneity and the vast majority of these cells show proliferative capacity even in late periods of development. In addition, we found that some marker of RGCs also labelled cells with a presumptive oligodendroglial nature.

The high proliferative capacity of cartilaginous fishes and also the persistent presence of RGCs postnatally makes them excellent candidates for study, not only the ancestral condition of neurogenic and/or gliogenic processes, but also other possible roles of RGCs during development and postnatal stages. This work would lay the foundation for future studies that investigate the proliferative potential and progeny of RGCs in basal vertebrates.

## Electronic supplementary material

Below is the link to the electronic supplementary material.

(**A**) Schema of the areas selected for cell counting, box area and distance between sections selected. (**B**) Table showing averages and standard deviations included in Figs. 7B, C, E, F. (**C, D**). Bar representations showing percentage of cells grouped by marker and area (TIF 35723 KB)

## Abbreviations

BLBP: Brain lipid binding protein
BSA: Basal superficial area
ChP: Choroid plexus
DMP: Dorsomedial pallium
DP: Dorsal pallium
Dpcm: Dorsal pallium centromedial pars
GFAP: Glial fibrillary acidic protein
GS: Glutamine synthase
Hab: Habenula
LGE-h: Lateral ganglionic eminence homologue
MGE-h: Medial ganglionic eminence homologue
MP: Medial pallium
OB: Olfactory bulb
P: Pallium
PCNA: Proliferating cell nuclear antigen
pLP: Presumptive lateral pallium
pMGE-h: Presumptive medial ganglionic eminence homologue
pMP: Presumptive medial pallium
PSB: Pallial–subpallial boundary
pVLP: Presumptive ventrolateral pallium
pVP: Presumptive ventral pallium
RGCs: Radial glial cells
SP: Subpallium
Spt: Septum
VLP: Ventrolateral pallium
